# Crystal structure of the CoV-Y domain of SARS-CoV-2 nonstructural protein 3

**DOI:** 10.1038/s41598-023-30045-9

**Published:** 2023-02-18

**Authors:** Yunfeng Li, Yulia Pustovalova, Wuxian Shi, Oksana Gorbatyuk, Sridhar Sreeramulu, Harald Schwalbe, Jeffrey C. Hoch, Bing Hao

**Affiliations:** 1 Department of Molecular Biology and Biophysics, University of Connecticut Health Center, 263 Farmington Avenue, Farmington, CT 06030-3305 USA; 2 Photon Sciences, Brookhaven National Laboratory, Upton, NY 11973 USA; 3 Institute for Organic Chemistry and Chemical Biology, Center of Biomolecular Magnetic Resonance (BMRZ), Goethe University Frankfurt, Frankfurt Am Main, Germany

**Keywords:** Biochemistry, Biophysics, Microbiology, Structural biology, Diseases

## Abstract

Replication of the coronavirus genome starts with the formation of viral RNA-containing double-membrane vesicles (DMV) following viral entry into the host cell. The multi-domain nonstructural protein 3 (nsp3) is the largest protein encoded by the known coronavirus genome and serves as a central component of the viral replication and transcription machinery. Previous studies demonstrated that the highly-conserved C-terminal region of nsp3 is essential for subcellular membrane rearrangement, yet the underlying mechanisms remain elusive. Here we report the crystal structure of the CoV-Y domain, the most C-terminal domain of the SARS-CoV-2 nsp3, at 2.4 Å-resolution. CoV-Y adopts a previously uncharacterized V-shaped fold featuring three distinct subdomains. Sequence alignment and structure prediction suggest that this fold is likely shared by the CoV-Y domains from closely related nsp3 homologs. NMR-based fragment screening combined with molecular docking identifies surface cavities in CoV-Y for interaction with potential ligands and other nsps. These studies provide the first structural view on a complete nsp3 CoV-Y domain, and the molecular framework for understanding the architecture, assembly and function of the nsp3 C-terminal domains in coronavirus replication. Our work illuminates nsp3 as a potential target for therapeutic interventions to aid in the on-going battle against the COVID-19 pandemic and diseases caused by other coronaviruses.

## Introduction

A characteristic feature of single-stranded positive-sense RNA viruses, which include the family *Coronaviridae* of the order *Nidovirales*, is their ability to restructure intracellular membranes to form double-membrane vesicles (DMVs) that serve as the central hubs for viral RNA replication^1,2^. Specifically, following virus entry, *Betacoronaviruses*, including human pathogens Severe Acute Respiratory Syndrome Coronavirus (SARS-CoV)^3,4^, Middle East Respiratory Syndrome Coronavirus (MERS-CoV)^5^ and the recently emerged SARS-CoV-2^6–8^, assemble a network of the viral replication-transcription complexes (RTCs) within the endoplasmic reticulum (ER)-transformed DMVs, effectively organizing viral genome replication by sequestering and localizing viral RNA replication proteins, viral RNA templates and essential host factors^1,2,9–12^. The RTC-bound DMVs provide a microenvironment suitable for viral RNA synthesis and offer a potential means to evade host cell defenses. Although the architecture of these rearranged membrane vesicles and their central role in viral replication have been well characterized in many positive-sense RNA viruses, the precise mechanism behind their formation and operation is still poorly defined^2,13^. In light of the unprecedented scale of the ongoing global outbreak of the COVID-19 pandemic, a detailed understanding of this critical step in the coronavirus life cycle is expected to have important implications for designing next-generation vaccines and antiviral therapeutics.

Significant efforts have been devoted to identifying viral factors essential for DMV formation. Two-thirds of the SARS-CoV-2 genome encodes two replicase polyprotein precursors that are subsequently auto-proteolytically processed into 16 nonstructural proteins (nsp1-16)^1,14^. Three nsps, nsp3, nsp4 and nsp6, contain transmembrane domains and thus are the prime candidates for directing DMV formation. Indeed, previous studies have shown that co-expression of nsp3 and nsp4 of either SARS-CoV or MERS-CoV is required to induce ER membrane pairing and the curvature that eventually wrap a membrane segment into a DMV^15,16^. Moreover, the C-terminal region of SARS-CoV nsp3 together with the full-length nsp4 have been shown to be sufficient to trigger membrane rearrangement^17,18^. More recently, a cryo-electron tomography study of murine hepatitis coronavirus (MHV) has further expanded knowledge of the architecture of DMVs in their native state by discovering a cylindrical pore complex connecting the DMV interior with the cytoplasm, possibly allowing release of newly synthesized RNA products^9^. Importantly, it was found that six copies of nsp3 form a crown-like structure on the cytoplasmic side of the pore, supporting the notion that nsp3 is the core component of the viral replication machinery.

**Figure 1:**
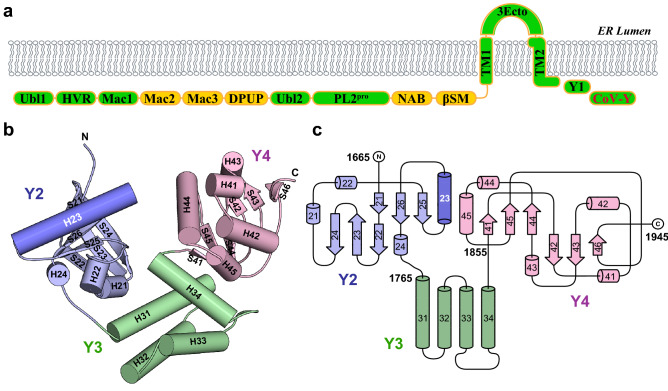
Overall structure of the CoV-Y domain of SARS-CoV-2 nsp3. (**a**) Domain organization of SARS-CoV-2 nsp3. The ten domains conserved in all coronaviruses are colored in green. The full names of the domains and their amino acid residue boundaries are listed in Supplementary Table S1. The CoV-Y construct used in this study is described in Supplementary Fig. S1. (**b**) Ribbon diagram of CoV-Y, with secondary structure elements labeled. The three subdomains, Y2, Y3 and Y4, are shown in purple, green and pink, respectively; this color scheme is maintained throughout the manuscript unless specified. The H23 helix is shown in dark purple. (**c**) Topology diagram of CoV-Y. Cylinders and arrows represent helices and β strands, respectively.

Nsp3 is the largest protein encoded by any known coronavirus genomes (1,945 amino acid residues in SARS-CoV-2) and possesses multiple domains with a variety of functions^19,20^ (Fig. 1a and Supplementary Table S1). In addition to its role in DMV biogenesis, polyprotein autoprocessing and host immune evasion, nsp3 acts as a membrane-anchored scaffold recruiting coronaviral and cellular proteins, and possibly RNA, to initiate the assembly of RTCs in infected host cells^21^. Nsp3 can be divided into three parts, including a large N-terminal cytosolic region (~ 157 kDa in SARS-CoV-2 with ten distinct domains) followed by a transmembrane region (~ 20 kDa with three domains) and a C-terminal cytosolic region (~ 41 kDa with two domains). Each of the N-terminal domains of nsp3, except the glutamic-acid-rich domain (HVR) that is likely intrinsically disordered and the Mac3 domain that is presumably highly homologous to Mac1 and Mac2, have been structurally characterized in detail by X-ray crystallography or nuclear magnetic resonance (NMR) spectroscopy^22–24^ (Supplementary Table S1). On the other hand, little is known about structures of the transmembrane and C-terminal regions of nsp3 or their exact function in the viral replication cycle. This part of nsp3 consists of two transmembrane domains (TM1 and TM2) separated by a luminal ectodomain (3Ecto) and two Y domains (referred to as Y1 and CoV-Y) in the C terminus (Fig. 1a). The current working model proposes that TM1 and TM2 anchor nsp3 to the ER membrane so that the interactions between the ectodomains of the neighboring nsp3 and/or nsp4 induce membrane pairing and curvature, a prerequisite for DMV formation^18^. As one of the most conserved coronavirus-specific domains in nsp3^19,20^, however, the structure and function of the Y1 and CoV-Y domains remain to be elucidated.

In an effort to understand the role of the nsp3 Y domains in the coronavirus replication cycle, we have determined the crystal structure of the 286-residue CoV-Y domain of the SARS-CoV-2 nsp3, the first complete CoV-Y domain structure for any known coronavirus. The 2.4 Å-resolution structure reveals that CoV-Y possesses a previously uncharacterized V-shaped fold consisting of three distinct subdomains named Y2, Y3 and Y4. Both sequence alignment and three-dimensional (3D) structure prediction suggest that this fold is likely shared by the CoV-Y domains from different coronavirus strains. Using large-scale NMR-based fragment screening followed by molecular docking, we identify cavities in CoV-Y that have the capacity for interactions with specific ligands or other nsps. Our structural analyses support the hypothesis that the Y1 and CoV-Y domains together are a part of the DMV pore complex and engage in specific interactions with the membrane bilayer.

## Results

### Protein production and structure determination

To enable structural study of the C-terminal domains of the SARS-CoV-2 nsp3, we designed multiple CoV-Y-containing constructs and examined their protein expression level as well as folding propensity and stability. We previously described a protocol for expression and purification of a CoV-Y-containing construct (residues 1638–1945) that exhibits well-dispersed signals in NMR spectra consistent with a folded protein^25^. Still, this construct has a largely unstructured N terminus, evidenced by a high degree of spectral crowding in the central region of the two-dimensional (2D) ^1^H–^15^N-TROSY spectrum. As a result, a shorter construct (residues 1660–1945) was designed and used for subsequent NMR and X-ray crystallography studies (Supplementary Fig. S1). This construct was expressed in *Escherichia coli* and the resulting recombinant protein is monomeric in solution as determined by size-exclusion chromatography (Supplementary Fig. S1c). The ^1^H–^15^N TROSY spectrum of the protein exhibits an extremely well-dispersed set of resonances, consistent with a monomeric protein of ~ 32 kDa, and nearly complete backbone assignment was achieved^26^. We also succeeded in obtaining diffraction-quality crystals of this construct. The CoV-Y crystals contain one protein molecule per asymmetric unit. The structure was determined and refined at a resolution of 2.4 Å by multiple-wavelength anomalous dispersion using data collected from a selenomethionine-substituted crystal (Table 1). The completed electron density map allowed unambiguous tracing of most of the protein except the five N-terminal residues (residues 1660–1664) (Supplementary Fig. S2). Crystal packing analyses of the symmetry-related molecules confirm that the CoV-Y construct used in this study is a monomer (Supplementary Fig. S3). The final structure was refined to an R factor of 19.2% and an R_free_ of 23.2% with good stereochemistry (Table 1).

**Table 1:** Summary of crystallographic analysis.

	Nsp3 CoV-Y	SeMet-MAD (peak)	SeMet-MAD (inflection)
Data collection			
Wavelength (Å)	0.97893	0.97908	0.97924
Space group	*P65*	*P65*	*P65*
Cell dimensions (Å)			
* a*, *b*, *c* (Å)	109.4,109.4, 61.2	109.3, 109.3, 60.8	110.1,110.1, 61.1
* α*, β, γ (°)	90, 90, 120	90, 90, 120	90, 90, 120
Resolution (Å)	19.9–2.4 (2.5–2.4)	29.0–2.7 (2.8–2.7)	29.0–2.7 (2.8–2.7)
* R*_sym_ (%)	4.4 (181.0)	14.2 (97.8)	13.7(100.2)
Mean (*I*/σ*I*)	31.6 (2.2)	19.8 (2.9)	20.00 (3.9)
CC_1/2_	1.0 (0.9)	1.0 (0.9)	1.0 (1.0)
Completeness (%)	97.5 (99.9)	97.2 (63.5)	99.5 (94.2)
Redundancy	19.5 (21.0)	30.1 (23.9)	32.8 (29.3)
Refinement			
Resolution (Å)	19.9–2.4		
No. reflections (|F| > 0σ)	15,488		
* R*_work_/*R*_free_ (%)	19.2/23.2		
No. atoms			
Protein	2164		
Water	48		
Average B-factors (Å^2^)			
Protein	103.418		
Water	98.597		
R.m.s. deviations			
Bond lengths (Å)	0.010		
Bond angles (°)	1.19		

Values in parentheses are for the highest-resolution shell. *MAD* multiple wavelength anomalous dispersion.

### Overall structure of the nsp3 CoV-Y domain

**Figure 2:**
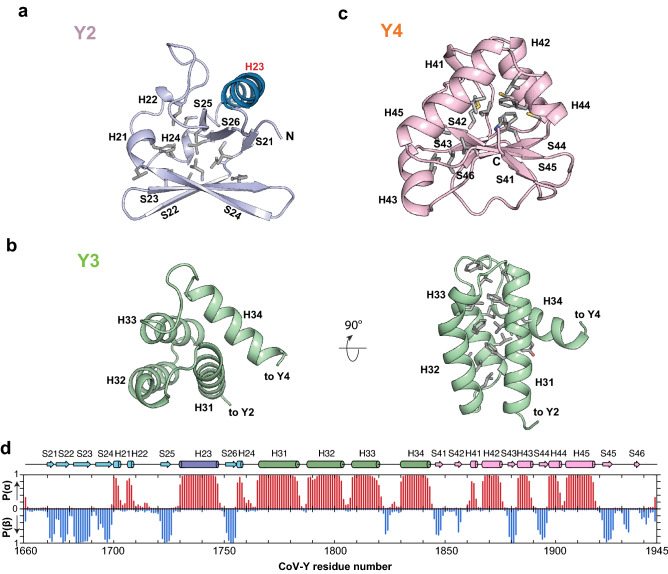
The CoV-Y domain comprises three distinct subdomains. (**a**) Ribbon diagram of the Y2 subdomain. Residues lining the internal hydrophobic core are shown in stick representation. (**b**) Ribbon diagram of the Y3 subdomain. A cluster of hydrophobic and aromatic residues in the central core of the helix bundle is shown in stick representation. (**c**) Ribbon diagram of the Y4 subdomain. The hydrophobic residues in its core are shown in stick representation. (**d**) Secondary structure propensity of the CoV-Y domain in solution derived using TALOS-N^58^ as shown previously^26^. Probabilities of occurrence for helices [P(α)] and β-sheet [P(β)] are shown in red and blue, respectively. The corresponding secondary structure elements from the X-ray structure of the CoV-Y domain are shown across the top as cylinders (helices) and arrows (β strands).

The CoV-Y domain of SARS-CoV-2 nsp3 has a twisted structure resembling a letter V with linear dimensions of approximately 60 Å × 50 Å × 32 Å (Fig. 1b,c). The protein is organized into three distinct subdomains (Y2, Y3 and Y4) with a deep cleft in the middle; previous proteomic analysis suggested that the CoV-Y domain of SARS-CoV nsp3 consists of two subdomains^27^. The N-terminal Y2 subdomain (residues 1665–1763) is dominated by six β strands (S21–S26) arranged into two nearly orthogonal β sheets (S21↑S26↑S25↑ on one sheet and S22↑S23↓S24↑ on the other) to form a β sandwich-like structure packed across a hydrophobic core (Fig. 2a). A 16-residue α helix (H23) connecting strands S25 and S26 stacks on the exposed side of the parallel sheet, whereas three short 3_10_ helices (H21, H22 and H24) surround the open side of the β sandwich.

The middle Y3 subdomain (residues 1764–1847) adopts a compact α helical fold composed of a three-helix bundle (H31–H33) packing against the 4th helix (H34) with an axial tilt of ~ 60° (Fig. 2b). The antiparallel helix bundle has a down-up-down topology, of which three helices interact over four turns and bury 2729 Å^2^, equivalent to 49% of their combined total accessible surface area. A cluster of aromatic side chains (Phe1773, Tyr1776, Phe1780, Phe1784, Phe1815 and Phe1823) forms a twisted ladder in the central core of the helix buddle stabilizing interactions between three helices. All four helices of the Y3 subdomain are largely amphipathic, with polar and charged residues on the outside and nonpolar side chains buried inside (Fig. 2b).

The C-terminal Y4 subdomain (residues 1848–1945) consists of a α/β fold with two three-stranded β sheets (S41↑S45↑S44↑ and S42↓S43↓S46↑) in the center flanked by three α helices (H42–H44) and a 3_10_ helix (H41) (Fig. 2c). The two β sheets run alongside each other with a tilt of ~ 30° and are stabilized mainly by electrostatic and side-chain hydrogen-bonding interactions between S44 and S42. Two helix pairs (H41/H42 and H44/H45) pack against one face of the β sheets to form a dome-like structure that is lined with a network of hydrophobic side chains in its core (Fig. 2c).

Among three subdomains, Y2 and Y3 are held together by a short linker and a polar interface between the antiparallel sheet of Y2 and the helix bundle of Y3. Y4 packs tightly against Y3 and the Y3–Y4 interface is dominated by hydrophobic interactions between H34 of Y3 and H44 and S45 of Y4. On the other hand, there is only minimal interaction between Y2 and Y4 with the side chains of Gln1769 (Y2) and Lys1909 (Y4) forming a salt bridge crossing the middle of the cleft. Importantly, the secondary structures of CoV-Y observed in the crystal structure are in excellent agreement with the secondary-structure elements predicted from the assigned backbone and C^β^ chemical shifts except for the extreme C-terminal region^26^, indicating that CoV-Y adopts a similar fold in solution (Fig. 2d).

### The monomeric structure of CoV-Y is unique

The overall structure of CoV-Y reveals a peculiar topology without a high-degree of similarity to any known fold in the Protein Data Bank (PDB). A hierarchical search against the PDB and the AlphaFold database using Dali^28^ failed to reveal any other proteins with a topology significantly similar to that of CoV-Y [Z-scores > 10; root-mean-square deviation (rmsd) < 10 Å]. Interestingly, a relatively remote similarity was found with a number of AAA + ATPase domain-containing proteins in various organisms (Z-scores ~ 7; rmsd ~ 10 Å). The characteristic feature of AAA + proteins is a structurally conserved ATP-binding module that assembles into oligomeric ring-like complexes in which large subdomains (AAA-LD) combine to form the AAA ring and the small subdomains (AAA-SD) form the protrusions as in a pinwheel^29^. The individual subdomains of CoV-Y can be superimposed rather poorly with two AAA domain pairs of *Schizosaccharomyces pombe* Mdn1, an AAA-containing protein essential for ribosome biogenesis^30^. The secondary-structure elements of Y2 and Y4 of CoV-Y show certain structural resemblance with the AAA-LD, whereas the helical Y3 subdomain shares a fair degree of similarity with the AAA-SD of Mdn1 (PDB ID: 6ORB); CoV-Y and Mdn1 share a 5.5% sequence identity and display a 5.4-Å rmsd for 109 corresponding C_α_ atoms (Supplementary Fig. S4a). Although the spatial arrangement of three CoV-Y subdomains is well aligned with two neighboring AAA-LDs and an associated AAA-SD of Mdn1, their respective hydrophobic cores and potential ligand binding pockets are quite distinct, indicating that an evolutionary relationship is unlikely.

We also performed Dali searches against the individual CoV-Y subdomains. For Y2, the β sandwich-like fold was found to have a marginal similarity with a part of the N-terminal domain of a *Bacteroides* spp. β-hexosaminidase^31^ (PDB ID: 6Q63; Z-score = 4.1; rmsd = 5.8 Å) (Supplementary Fig. S4b). Y3 shows low structural resemblance to the helical domain of ent-copalyl diphosphate synthase from *Arabidopsis thaliana* (PDB ID: 3PYB; Z-score = 6.4; rmsd = 3.0 Å) (Supplementary Fig. S4c). Likewise, Y4 does not exhibit significant similarity to other proteins; it can only be partially superimposed with the N-terminal domain of archaeal Ribonuclease P^32^ (PDB ID: 6K0B) with a low Z-score (3.3) and high rmsd (3.9 Å; Supplementary Fig. S4d). On the other hand, the fold of Y4 in CoV-Y is essentially identical to the crystal structure of Y4 determined independently (residues 1844–1943; PDB ID: 7RQG; Supplementary Fig. S4d). The two structures can be superimposed with a 0.7 Å rmsd in C_α_ positions, indicating that interaction with Y2 and Y3 subdomains does not cause significant conformational change in Y4. Taken together, we conclude that CoV-Y possesses three distinct subdomains and represents a previously uncharacterized type of protein fold that may have the potential to form larger assemblies.

### The structure of CoV-Y is conserved among human coronavirus homologs

All three coronaviruses that have caused the epidemic and pandemic outbreaks of diseases in human populations belong to *Betacoronaviruses* with SARS-CoV-2 and SARS-CoV being from *Sarbecovirus* subgroup and MERS-CoV from *Merbecovirus* subgenus (https://talk.ictvonline.org/ictv-reports)^33,34^. In addition, four other strains of coronaviruses have been identified to associate with mild upper respiratory diseases in immunocompetent humans, among which HCoV-OC43 and HCoV-HKU1 are classified in the *Embacovirus* subgroup of *Betacoronaviruses*, while HCoV-229E and HCoV-NL63 belong to *Alphacoronavirus*^33,34^. The nsp3 CoV-Y domains of four human *Betacoronavirus* strains have sizes (369–377 residues) nearly identical to that of the SARS-CoV-2 nsp3 CoV-Y (369 residues), whereas those of two *Alphacoronavirus* strains (HCoV-229E and HCoV-NL63) are smaller (345 residues; Supplementary Fig. S5). Recent studies of the SARS-CoV-2 viral genome have also identified two close relatives, one found in horseshoe bat (BatCoV-RaTG13)^7^ and another in pangolin (Pangolin-CoV)^35^; it has been suggested that these two viruses are the natural and intermediary hosts of SARS-CoV-2, respectively^7,34^.

**Figure 3:**
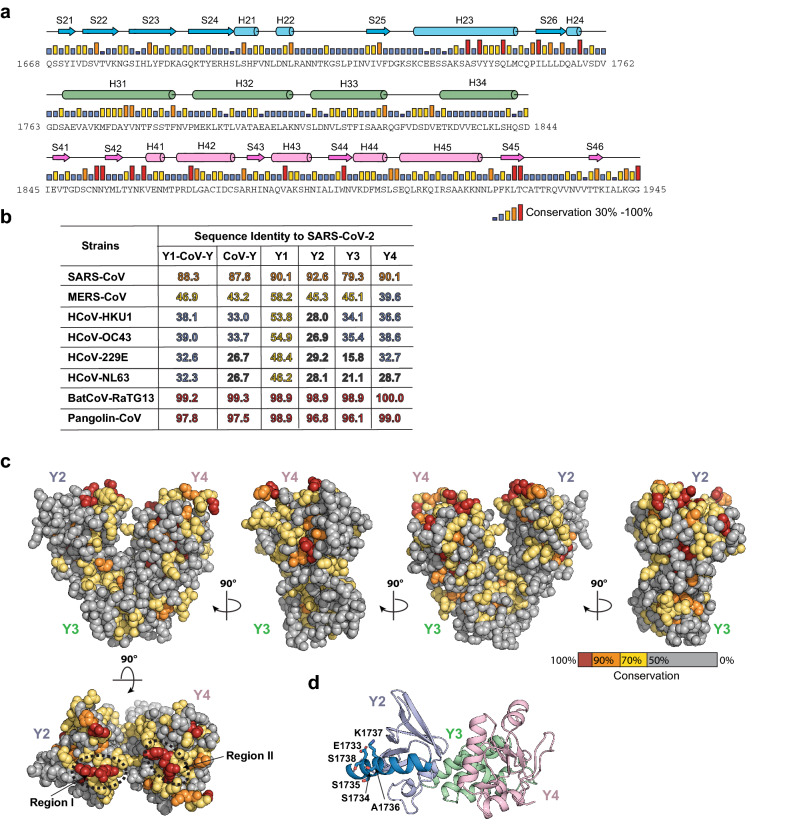
Sequence conservation among nsp3 CoV-Y homologs. (**a**) Sequence conservation of the nsp3 CoV-Y domain among seven human coronaviruses and two close relatives of SARS-CoV-2 (see “Methods” and Supplementary Fig. S5 for sequence information). Conservation is shown as a bar graph, with red bars indicating identity among nine CoV-Y homologs. Secondary-structure assignments of CoV-Y from the crystal structure are shown as cylinders (helices) and arrows (β strands). (**b**) Sequence identity of the Y1–CoV-Y, CoV-Y and four CoV-Y subdomains of the above eight coronavirus nsp3 sequences to those of SARS-CoV-2 nsp3. The ClustalW alignments were performed using DNASTAR Lasergene Suit 8. The identity numbers are shown in a color scale, with red indicating conservation above 95%. (**c**) Molecular surface of the CoV-Y domain structure colored according to homologous conservation. Dashed ellipse indicates the two highly conserved regions. (**d**) Close-up view of the H23 helix of Y2. The six residues with most variation in SARS-CoV-2 isolates are shown in stick representation.

Sequence alignments of the nsp3 CoV-Y domains from all nine coronavirus strains show that they share ~ 27–99% pairwise sequence identities (Fig. 3). Among SARS-CoV-2, BatCoV-RaTG13 and Pangolin-CoV, only eight nonsynonymous substitutions, most of which maintain hydrophobicity and polarity of the residue, are found in their respective CoV-Y domains, confirming a close relationship between these three strains (Supplementary Fig. S5). For the human coronavirus strains, the SARS-CoV-2 CoV-Y shares 88% sequence identity with that of SARS-CoV, whereas the other strains including MERS-CoV show a much more distant relationship with identities ranging from 32 to 47% (Fig. 3b). As for the individual subdomains, Y3 is the least conserved domain among human strains with sequence identities ranging from 16 to 79%, whereas Y2 and Y4 share relatively higher conservation (27% to 93%) (Fig. 3b and Supplementary Fig. S5). However, those highly conserved residues are located throughout the entire sequences of the CoV-Y domains (Fig. 3a), and it is not immediately clear whether they play any role in mediating nsp3 function.

**Figure 4:**
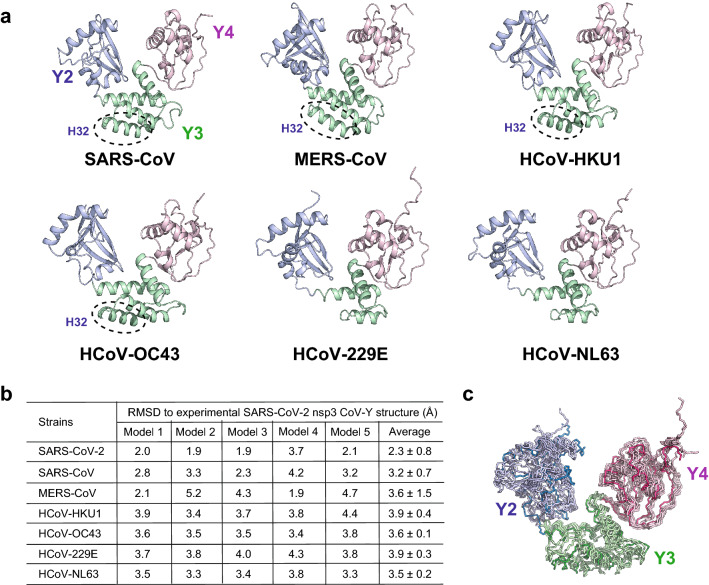
Structural conservation among nsp3 CoV-Y homologs. (**a**) Ribbon diagrams of the top-ranked AlphaFold-computed structural models of the nsp3 CoV-Y domains for four human *Betacoronaviruses* (SARS-CoV, MERS-CoV, HCoV-HKU1 and HCoV-OC43) and two human *Alphacoronaviruses* (HCoV-229E and HCoV-NL63). Dashed ellipse indicates the H32 helix of Y3 on the *Betacoronaviruses* that is presumably lacking in *Alphacoronavirus* CoV-Y. (**b**) The RMSDs of the five top-scored models calculated using AlphaFold^36^ to the crystal structure of the SARS-CoV-2 nsp3 CoV-Y. (**c**) Superposition of the crystal structure and six structural models of the CoV-Y homologs.

With the CoV-Y structure at hand, we can elaborate on several questions relating to sequence and structure conservation among its homologs. First, computationally-predicted 3D models suggest that the annotated Co-V domains of other human coronaviruses likely have structures similar to that of the SARS-CoV-2 CoV-Y (Fig. 4a). The overall fold of the six human CoV-Y homologs predicted using AlphaFold^36^ closely resembles the structure observed for the SARS-CoV-2 CoV-Y with a 3.2–3.9 Å rmsd for equivalent C_α_ positions. We note that AlphaFold is, in this instance, highly accurate in predicting a novel fold because the calculated SARS-CoV-2 CoV-Y structure can be superimposed with the crystal structure with a 2.3 ± 0.8 Å rmsd in C_α_ positions (Fig. 4b). This finding is significant in light of ongoing debates over the ability of machine learning to innovate beyond the domain of possibilities present in the training data. It is also striking that individual subdomains in the predicted structures share common topologies to those in the crystal structure (Fig. 4c). Major conformational differences lie in three extended loop regions connecting H22 and S25 in Y2, H33 and H34 in Y3, and H41 and H42 in Y4. Notably, the Y3 subdomains of the two *Alphacoronavirus* strains (HCoV-229E and HCoV-NL63) feature only three helixes and lack the presumed H32 helix found in all *Betacoronavirus* strains, consistent with the smaller sizes of their CoV-Y domains (Fig. 4a,c and Supplementary Fig. S5). In addition, all five top models calculated for each CoV-Y homolog are generally matched closely to each other, with rmsd ranging from 1.0 to 2.3 Å in C_α_ positions, suggesting that top-ranked models display good fidelity (Supplementary Table S2). These analyses suggest that the core fold of the SARS-CoV-2 nsp3 CoV-Y domain likely represents a common fold for all CoV-Y domains in known human coronaviruses.

Second, the investigation of homologous sequence conservation in the structural context of the CoV-Y domains identifies two most highly conserved surface areas (Regions I and II) in the nsp3 homologs (Fig. 3c). Region I is located on the C-terminal corner of the long helix H23 followed by the strand S26 in Y2, including four invariant residues, Ala1739/V1741/Gln1745 in H23 and Ile1751 in S26. Region II is located on the base of the two β sheets and their surrounding helices and loops in Y4, including eight invariant residues, Asn1853/Asn1854 (S42), Tyr1859/Lys1861 (the loop connecting S42 and H41), Asp1869 (H42), Trp1895 (S44) and Leu1924/Thr1925 (S45). These two regions have much higher sequence conservation than the rest of the structure, suggesting that they may play important roles in mediating specific functions of the nsp3s.

Last, as an RNA virus, SARS-CoV-2 is known to rapidly accumulate genomic mutations and some of these nonsynonymous amino-acid residue changes may have structural and functional impact on the encoded proteins^24,37^. Previous studies based on the analysis of nearly 200,000 individual SARS-CoV-2 viral genomes over the course of the ongoing COVID-19 pandemic have suggested that the C-terminal region of nsp3 is less frequently mutated compared to the other parts of the genome, likely due to its key role in inducing DMV formation^37^. To gain new insights into the distribution of naturally occurring nsp3 Y1–CoV-Y variations, we conducted a broad BLAST search of 5,000 individual SARS-CoV-2 isolates using the reference Y1–CoV-Y sequence (residues 1577–1945; NCBI Accession #: YP_009742610) as a query. Sequence alignments of the Y1–CoV-Y domains show that they share ~ 99.46–100% pairwise sequence identities, in which 360 sequences have one variation and one sequence has two variations. Remarkably, the entirety of the 362 variations are located among six residues in the middle of the H23 helix in the Y2 subdomain, ranging from Glu1733 to Ser1738 (Fig. 3d and Supplementary Fig. S5). Among six variants, A1736V was detected 355 times (~ 98%), S1738L three times and the rest including E1733G, S1734P, S1735F and K1737R once. Since these amino acid substitutions were likely found in human isolates, we infer that those nsp3 CoV-Y variants correspond to stable, functional proteins. We note that five of the substitutions except K1737R increase the hydrophobicity of the respective residue. In addition, E1733G and S1734P are two amino acid substitutions that may disrupt the helix. As discussed above, the entire H23 helix is exposed in CoV-Y, yet it is predicted to interact extensively with a β hairpin in Y1 (see below). S1738L would break an observed hydrogen bond with Ile1608 of Y1, while Ser1735/Ala1736/Lys1737 makes no contact with Y1. Whether those substitutions play any role in mediating Y1–CoV-Y function and/or its interactions with other proteins requires further investigation.

### Fragment screening of CoV-Y

**Figure 5:**
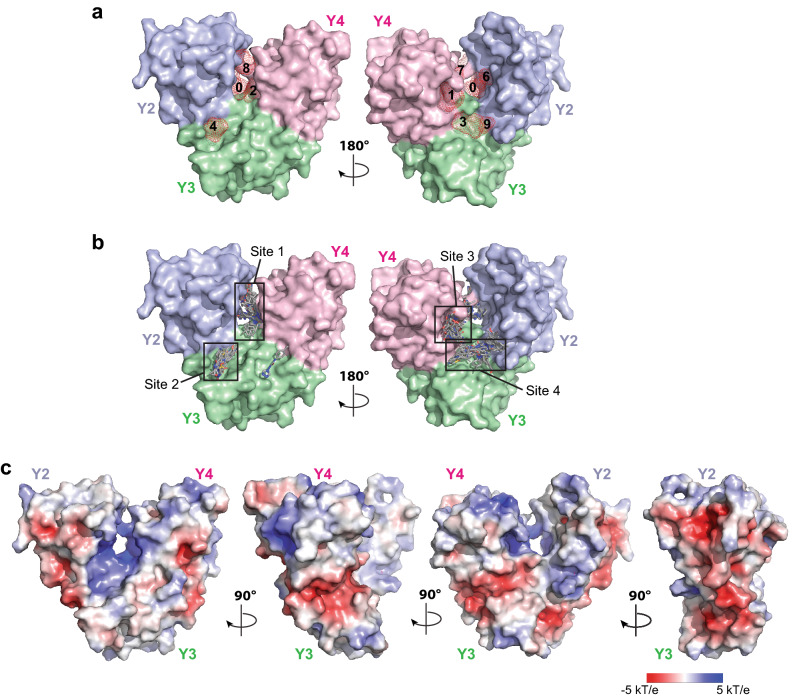
Molecular docking of the NMR-identified binders onto the structure of CoV-Y. (**a**) Molecular surface of CoV-Y showing the ten surface cavities identified using the FTMap server (https://ftmap.bu.edu). (**b**) Molecular docking of the 17 binders identified by NMR fragment-screening onto the CoV-Y structure by AutoDock Vina^57^. The top-ranked docking pose of each binder is shown in stick representation in four sites overlapped with those identified by FTMap. Chemical structure of each binder and the estimated binding energy of their top docking poses are listed in Supplementary Table S3. (**c**) Molecular surface representation of CoV-Y, colored according to the local electrostatic potential ranging from − 5 kT/e in deep red (most negative) to 5 kT/e in dark blue (most positive), calculated using the program ABPS^59^.

To gain insights into functions of the nsp3 CoV-Y domain, we partnered with the international COVID19-NMR consortium (https://covid19-nmr.de/) in a large-scale NMR-based ligand screening campaign that aimed to identify poised fragments targeting SARS-CoV-2 proteins^38^. The DSI (Diamond-SGC-iNEXT)-poised library consisting of 768 fragments represents a small collection of diverse compounds (< 300 Da) that bind promiscuously but cover a large chemical space and facilitate streamlined downstream hit optimization^39^. Four different ^1^H-based NMR experiments of ligands, including chemical shift perturbation, waterLOGSY, saturation transfer difference and differential *T*_2_-relaxation, in the presence and absence of the unlabeled CoV-Y protein were recorded and analyzed^38^. In total, 81 displayed changes in at least one of the four NMR experiments. Among them, 17 fragments were qualified as binders for CoV-Y based on the spectral changes observed in at least two out of four NMR experiments (Supplementary Table S3). Following the computational mapping strategy developed by the consortium^38^, we next employed FTMap^40^ to identify accessible surface cavities in the CoV-Y structure. Using 16 small organic compounds provided by the FTMap server as scanning probes, we identified ten ligand-binding hot spots (clusters 0–9) that are clustered largely around the deep cleft in the middle of the V-shaped structure crossing the interfaces between Y2/Y3 and Y2/Y4 (Fig. 5a).

We next used molecular docking to locate the potential surface cavities on CoV-Y capable of accommodating the identified fragment binders. Each of the 17 fragments was docked to CoV-Y as a flexible entity using AutoDock Vina^41^; the binding energies of the top poses of each binder range between − 4.7 kcal/mol and − 6.4 kcal/mol (Supplementary Table S3). As expected, most of the top-ranked poses of the fragments dock into the same cavities identified by FTMap, which can be organized into four distinguishable sites (Fig. 5b). Site 1 sits in the center of the Y2/Y3/Y4 interface and is surrounded by three helices (H23, H34 and H44) from the three subdomains; this site overlaps with the FTMap clusters 0, 2 and 8. Site 2 is located in a shallow surface groove between the H22 of Y2 and the helix bundle of Y3, and crosses the FTMap cluster 4. Site 3 is a cavity formed by H44 and the central β sheet of Y4 and situated on the back of the site 1 close to the FTMap cluster 1. Site 4 is adjacent to the site 3 and located in a pocket formed by the outside β sheet of Y2 and the H31 and H34 of Y3; this site spans across the FTMap clusters 3 and 9. Interestingly, the cavities accommodating the sites 1, 2 and 4 are mostly positively charged, whereas the site 3 has a largely electronegative surface (Fig. 5c). However, we should point out that we do not have any known ligands or knowledge of the ligand binding site in CoV-Y, making it difficult to confirm the validity of the docking results. Additionally, docking programs can be sensitive to the geometrical structure of the target protein and the size of the ligand, making it challenging to compare the absolute free energy values among identified hits. Nevertheless, the goal of this high-throughput fragment-based screening method was to identify potential hits as a starting point for chemistry exploration and optimization. Thus, it is expected that the binding energy of the initial hits will be weak and will require further optimization to increase binding strength. Taken together, our structural and fragment binding data suggest that CoV-Y has a unique structure that can bind to specific ligands, which can be used to further functional studies and therapeutic development.

### Assembly of the Y1 and CoV-Y domains

**Figure 6:**
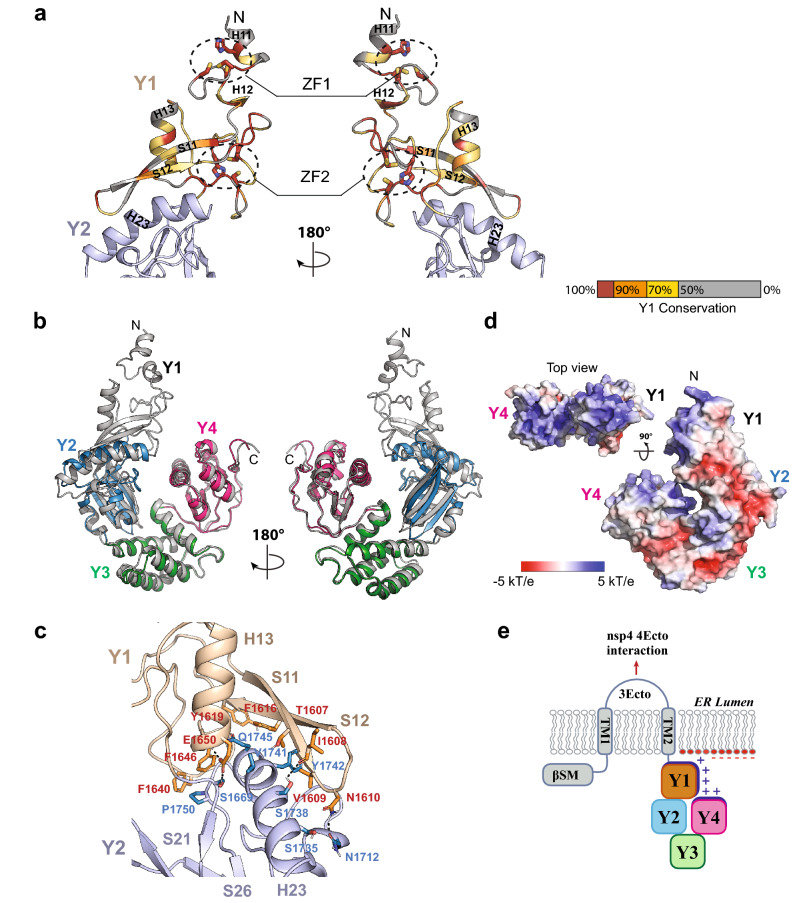
Assembly of the Y1 and CoV-Y domains. (**a**) Ribbon diagrams of the top-ranked AlphaFold-computed structural model of the SARS-CoV-2 nsp3 Y1 domain, colored according to homologous conservation as described in Fig. 3a. The coordinating residues of ZF1 (His1581, Cys1586, Cys1591 and Cys1594) and ZF2 (Cys1627, H1630, Cys1634 and Cys1637) are shown in stick representation. (**b**) Superposition of the crystal structure of CoV-Y and the top-ranked computed structural model of Y1–CoV-Y of SARS-CoV-2 nsp3. The CoV-Y structure is colored as in Fig. 1b, while Y1–CoV-Y is in gray. (**c**) Close-up view of the modeled Y1-Y2 interface showing interacting residues of Y1 (orange) and Y2 (blue). Hydrogen bonds are shown as black dashed lines. (**d**) Molecular surface representation of Y1–CoV-Y, colored according to the local electrostatic potential ranging from − 5 kT/e in deep red (most negative) to 5 kT/e in dark blue (most positive), calculated using the program ABPS^59^. (**e**) A schematic model showing the interaction between the nsp3 Y1–CoV-Y region and the subcellular membrane. We propose that the observed positively-charged (+) patch on the top face of Y1 and Y4 directly interacts with the anionic glycerophospholipids (−) embedded in the membrane and contribute to the formation of the transmembrane pore in DMVs.

The extreme C-terminal region of nsp3 contains the Y1 domain (residues 1577–1659) preceding the CoV-Y domain. The Y1 domain is predicted to be conserved in the majority of the viral families of the order *Nidovirales*, while CoV-Y is suggested to be present only in coronaviruses^19,20^. Indeed, SARS-CoV-2 nsp3 Y1 shares ~ 46–90% pairwise sequence identity in human coronaviruses, which is higher than those of the CoV-Y subdomains (Fig. 3b and Supplementary Fig. S5). Because we were unable to obtain diffraction-quality crystals of the construct comprising both Y1 and CoV-Y of SARS-CoV-2 nsp3, the Y1–CoV-Y structure was computed using AlphaFold^36^ (Fig. 6a,b). The predicted Y1 structure exhibits a novel fold highlighted with two adjacent zinc finger (ZF)-like motifs (Fig. 6a). The first ZF motif (ZF1) adopts a HCCC-type TAZ2 domain-like zinc-binding site^42^ formed by the C-terminus of the H11 helix (His1581), a short loop (Cys1586 and Cys1591) and the N-terminus of the H12 helix (Cys1594). The second ZF motif (ZF2) harbors a CHCC-type zinc-binding site in which the four ligands (Cys1627, His1630, Cys1634 and Cys1637) are located entirely in a short zinc-binding loop between the strand S12 and the helix H13. The eight zinc coordinating residues in ZF1 and ZF2 are invariant in all human coronaviruses (Supplementary Fig. S5), however, the biological roles of these two ZF motifs are presently unknown.

In the calculated Y1–CoV-Y structure, Y1 sits on the top of Y2 and interacts almost solely with Y2, except for a single hydrogen bond between Asn1638 of Y1 and Lys1917 of Y4 (Fig. 6b). We note that the predicted top five CoV-Y models in Y1–CoV-Y are highly similar to the crystal structure with an average 2.8 ± 1.4 Å rmsd in the equivalent C_α_ positions and a five-model variance of 1.5 ± 0.7 Å. The assembly of Y1 and Y2 is underpinned by their strong surface complementarity. The long β hairpin and the loop connecting S12 and H13 of Y1 form a surface groove in the shape of a car seat to embrace the H23 helix of Y2. The Y1-Y2 interaction is mediated by a network of hydrogen-bond and van der Waals contacts (Fig. 6c). At least three pairs of hydrogen bonds, Ile1608 (Y1)/Ser1738 (Y2), Asn1610 (Y1)/Asn1712(Y2) and Glu1650 (Y1)/Ser1669 (Y2), have been observed in all five top scored models. Additionally, a group of aromatic residues in Y1 (Phe1616, Tyr1617, Tyr1619, His1630, Phe1640 and Phe1646) and Y2 (Tyr1742, Tyr1743 and Pro1750) are packed around the interface. The association of Y1 and Y2 buries a total of ~ 1,530 Å^2^ of solvent-accessible surface on the two subdomains. Interestingly, a number of lysine and arginine residues, 11 from Y1 and 12 from Y4, line the top face of Y1 and Y4 to form a continuous positively-charged patch, while the large part of Y2 and Y3 are mostly negatively charged (Fig. 6d). These surface charge distributions might be involved in orienting the Y1–CoV-Y domains in such a way that the electropositive regions are directed toward and interact with the electronegative membrane surface (Fig. 6e).

We next used AlphaFold to compute the structural models of the Y1–CoV-Y domain of the six other human coronavirus nsp3s. As expected, all calculated models conformed to a conserved overall fold for Y1 as well as the arrangement of the four subdomains (Supplementary Fig. S6). Importantly, the structural conservation among those homolog proteins further reflected in the general similarities of the electrostatic surface of their subdomains. In particular, a significant portion of the exposed surface area of the Y1/Y4 subdomains in those homologs is electropositive, similar to that of the SARS-CoV-2 nsp3 Y1–CoV-Y (Supplementary Fig. S6a). These observations imply that specific membrane binding might be a conserved activity of the Y1–CoV-Y domain of nsp3 across the human coronavirus family.

### Implications for SARS-CoV-2 replication and coronavirus biology

The COVID-19 pandemic has highlighted the need for deeper knowledge of coronavirus biology for the development of novel intervention strategies. A complete characterization of virion components comprising the RTC is therefore important for understanding the dynamics and progress of the viral replication cycle. The mechanisms by which the viral nsps and host cell factors are recruited and localized to the defined subcellular locations to establish RTCs remain a longstanding unanswered question. As a key component of the RTC, significant progress has been made in characterization of nsp3 domains that possess well-conserved functions and activities. The C-terminal region of the protein, however, remains enigmatic. The sequence of this part of the protein. including the region from the first transmembrane helix to the C terminus of nsp3, does not show much similarity to other viral or cellular proteins. While it is suggested that the transmembrane region and the ectodomain in between comprise a membrane-anchoring platform essential for DMV membrane rearrangement^18^, the exact function of the Y domains is unclear. The goal of this study was to elucidate the structural properties of the CoV-Y domain of the SARS-CoV-2 nsp3 at the atomic level. Our results support a model in which the nsp3 Y domains are directly involved in the formation of the molecular pore in DMVs and perhaps mediate interaction with specific membrane lipids.

The crystal structure of the CoV-Y domain reveals an unusual configuration in which the three distinct subdomains form a unique V-shaped fold that has not been observed previously. The CoV-Y construct used in our study is monomeric in solution as determined by size exclusion chromatography, NMR and crystal packing analyses. However we note that the organization of three Co-Y subdomains is somewhat reminiscent of the assembly of the two conserved AAA + domains into a ring-like oligomers^29^. The recent electron microscopy study showed that molecular pores involving six copies of nsp3 span across DMV membranes in MHV-infected cells with the N-terminal nsp3 domains pointing towards the cytosol^9^. It is thus tempting to speculate that in addition to the transmembrane domains, the Y domains including Y1 and CoV-Y may form higher-order oligomeric complexes and play a role in pore formation. It is possible that the Y1 domain is required for assembly of the oligomeric Y1–CoV-Y complex. Despite expressing a number of different Y1–CoV-Y constructs, we were unable to produce homogeneous proteins that could be used for structural and biophysical studies. Therefore, at present we do not have sufficient evidence to definitively support a hexameric configuration for the nsp3 Y domains.

Both the sequence alignments and 3D structure prediction analyses suggest that the unique fold of CoV-Y represents the first structural view of the entire domain and is likely conserved among the CoV-Y homologs in coronaviruses. Furthermore, two specific sequence-conserved regions in the CoV-Y crystal structure, including the C-terminal portion of the H23 helix in the Y2 subdomain, have been identified that prioritize testing of the functions of the conserved side chains in vivo by site-directed mutagenesis. Interestingly, preliminary comparative sequence analysis of the genomes of the individual SARS-CoV-2 isolates reveals that all the naturally occurring variations in the Y1–CoV-Y region are clustered in the middle of the H23 helix of Y2. Notably, the H23 helix is also likely involved in the interaction with the Y1 domain based on the predicted Y1–CoV-Y structure. Together, those independent observations suggest that the H23 helix of Y2 or the CoV-Y domain as a whole may provide a framework for elucidating the correlation between viral evolution and constraints imposed by the structure and functionality of the encoded protein.

Analysis of the computed Y1–CoV-Y structure suggests that Y1 and Y4 form a continuous positively-charged top surface that in theory might potentially interact with headgroups of anionic glycerophospholipids in cellular and organelle membranes in eukaryotes^43–45^. Anionic phospholipids include phosphatidic acid, phosphatidylserine, phosphatidylinositol, and various forms of phosphatidylinositol phosphates^45^. These lipids are present in low abundance in eukaryotic membranes but are abundant in endomembrane compartments including ER and Golgi^43^. It is thus possible that in addition to the TM1–3Ecto–TM2 region, the Y domains of nsp3 may forecast potential orientations of the nsp3 protein by docking onto the ER plasma membrane through interactions with specific phospholipids (Fig. 6e).

In addition, our fragment screening and molecular docking studies have identified several exposed surface cavities in CoV-Y that provide valuable information for identifying potential ligand interaction and functional annotation. Moreover, previous studies have shown that the Y1 and CoV-Y domains interact not only with other domains (NAB and βSM that precede TM1) of nsp3 but other nsps (nsp9 and nsp12)^46^. A more recent study of the host interactomes of nsp3 has suggested that the 3Ecto–TM2–Y1–CoV-Y region of nsp3 interacts with host proteins associated with the ER-associated protein degradation pathways and proteins involved in cholesterol, lipid and N-glycan biosynthesis^21^. How those observed protein–protein/ligand interactions affect the respective function of nsp3 and other proteins remain unknown. Clearly, further study is required to determine the precise role of the nsp3 Y domains in viral replication and life cycle as a whole.

## Methods

### Protein expression and purification

The nsp3 CoV-Y construct (residues 1660–1945; 31.6 kDa; NCBI Accession #: YP_009742610) was produced as described^26^. In brief, the gene sequence was *Escherichia coli* codon optimized and cloned into a pET28a vector containing a removable tobacco etch virus (TEV) protease recognition site following the N-terminal His_6_ tag. (also see Supplementary Fig. S1a) CoV-Y was expressed in *E. coli* BL21(DE3) and purified by Ni^2+^-nitriloacetic acid affinity chromatography. After removal of the N-terminal His_6_ tag by overnight cleavage with TEV protease, the protein was further purified by anion-exchange (Source Q; GE Healthcare) and size-exclusion chromatography [Superdex 200 Increase 10/300 GL, GE Healthcare; equilibrated in a buffer containing 15 mM Hepes (pH 6.8), 75 mM lithium bromide, and 5 mM dithiothreitol (DTT)]. For crystallization, the protein was concentrated to 0.63 mM by ultrafiltration in the same buffer used for the size-exclusion chromatography. SeMet-substituted CoV-Y was produced following established procedures^47^ and purified as the wild-type protein. For ligand-observed ^1^H-NMR experiments, the wild-type unlabeled CoV-Y was expressed, purified and concentrated to 0.35 mM in a buffer containing 20 mM MOPS (pH 6.4), 100 mM lithium bromide, and 2 mM DTT.

### Crystallization and structure determination

CoV-Y was crystallized in a solution consisting of 0.2 M ammonium citrate, 20–30% polyethylene glycol 3350 at 4 °C using the hanging-drop vapor diffusion method. Crystals were cryoprotected in reservoir solution supplemented with glycerol to a final concentration of 18% and flash-cooled in liquid nitrogen. Diffraction data were collected at beamlines 17-ID-2 of National Synchrotron Light Source II (NSLS-II) and 12–2 and 9–2 of Stanford Synchrotron Radiation Lightsource (SSRL). The diffraction data were processed with autoPROC^48^ and Fast DP^49^. The crystals contain one molecule in the asymmetric unit. The structure of CoV-Y was determined by multiple-wavelength anomalous dispersion using the data collected at the selenium peak and inflection wavelengths. Seven independent selenium sites were located by HySS as implemented in AutoSol/PHENIX^50^, and initial phases calculated from these sites were improved by density modification using Phaser/PHENIX^51^. The resulting electron density map was readily interpretable and used to build more than 90% of the molecule with the program COOT^52^. Iterative cycles of refinement using REFMAC5/CCP4^53,54^ and BUSTER (Global Phasing Limited) followed by manual rebuilding in COOT were carried out until no further improvement of the R_free_ factor was observed. X-ray data collection and phasing and refinement statistics are summarized in Table 1. Ramachandran statistics of the final model were calculated using MolProbity^55^, in which 95.7% of the residues are in the favored region, 4.3% in the general allowed region, and none in the disallowed region. Molecular graphics were rendered using PyMOL (Schrödinger LLC).

### BLAST search, sequence alignment and computed 3D structural models

An initial multiple sequence alignment of the Y1 and the CoV-Y domains of the SARS-CoV-2 nsp3 (residues 1577–1945; NCBI Accession #: YP_009742610) was produced using the NCBI BLAST web server (https://blast.ncbi.nlm.nih.gov/Blast.cgi) to identify amino acid residue variations among 5,000 individual SARS-CoV-2 isolates. A ClustalW alignment of nsp3 Y1–CoV-Y coronavirus homologs was performed using DNASTAR Lasergene Siote 8 with the default settings. Annotations and region boundaries displayed in Supplementary Fig. S5 were manually fine-tuned to reflect predicted secondary or 3D structures. Computed structural models for nsp3 CoV-Y or Y1–CoV-Y and their human coronavirus homologs were generated using AlphaFold^36^, which was made available through NMRbox^56^ using servers equipped with NVIDIA A100 GPUs. Five models were calculated for each protein sequence and only the top-ranked model was presented in figures.

In addition to that of the SARS-CoV-2 nsp3 Y domains, the following sequences were used for Y1–CoV-Y homolog sequence alignments and 3D model calculation presented in Figs. 3 and 4, and Supplementary Fig. S5: SARS-CoV (residues 2372–2740; UniProt Accession #: P0C6U8), MERS-CoV (residues 2364–2740; UniProt Accession #: K9N638), HCoV-HKU1 (residues 2471–2840; UniProt Accession #: P0C6U3), HCoV-OC43 (residues 2382–2750; UniProt Accession #: P0C6U7), HCoV-229E (residues 2141–2485; UniProt Accession #: P0C6U2), HCoV-NL63 (residues 2119–2463; UniProt Accession #: P0C6U6), BatCoV-RaTG13 (residues 2394–2762; UniProt Accession #: A0A6B9WIQ1), and Pangolin-CoV (residues 2388–2756; UniProt Accession #: A0A6M3G868).

### Fragment screening, FTMap analysis and molecular docking

Ligand-based fragment screening by NMR spectroscopy using unlabeled CoV-Y was conducted previously at BMRZ at the Goethe University in Frankfurt, Germany as described^38^. The DSi-Poised library with 768 diverse fragments was screened in 64 mixes each containing 12 compounds as described previously^39^. In brief, each compound and CoV-Y were mixed in a ratio of 20:1 with a final concentration of the fragment at 200 μM and the protein at 10 μM. The ^1^H 1D spectra of the ligands were recorded on a 600 MHz spectrometer at 25 °C. Binding of the compounds to the protein was evaluated by four different ^1^H-based NMR measurements in the presence and absence of the protein: chemical shift perturbation, waterLOGSY effects, saturation transfer differences and signal intensity change in *T*_2_-relaxation experiments. In total 17 compounds were identified as CoV-Y binders based on the spectrum changes observed in at least two out of four NMR experiments^38^ (Supplementary Table S3). The surface cavities of CoV-Y were analyzed using the default settings at the FTMap web server^40^ (https://ftmap.bu.edu/login.php).

Docking of the fragments into the CoV-Y structure was performed using the iterated Local Search Global Optimization algorithm provided by AutoDock Vina^57^. The PDBQT format files (required as input) of both the fragments and CoV-Y were generated using the AutoDock Tools package provided by AutoDock 4. The flexible ligand docking protocol was adopted in this work to allow rotation of any rotatable bonds in the fragment when it is docked to the binding site in the protein. Once the rotatable bonds are identified, the fragment is allowed to be flexible by moving different rigid parts through the iterated local search global optimizer. During the global energy optimization, the ligand binding score is evaluated by an empirical scoring function, which is weighted sum of terms representing van der Waals, hydrogen bonds, electrostatic and desolvation contributions. The entire surface of the CoV-Y structure was searched for possible binding sites without bias. All other parameters were set at the default values defined by AutoDock Vina. In particular, a cutoff distance of 5 Å for van der Waals interactions and 3.7 Å for hydrogen bonds between ligand atoms and protein accepters were used. The default setting of exhaustiveness allows one hundred independent docking runs were performed for each fragment. The resulting poses were scored based on the free energy of binding (∆G) in which the least energetic poses would be ranked as the top hits. The estimated binding energy for the most favorable binding conformation of each docked fragment is listed in Supplementary Table S3.

## Supplementary Information

Supplementary Information.

## Data Availability

The atomic coordinates and structure factors generated during the current study are available in the RCSB Protein Data Bank (https://www.rcsb.org/) under Access Code 8F2E.
