# Supplementary material for: Crystal structure of the CoV-Y domain of SARS-CoV-2 nonstructural protein 3
Source: Sci Rep. 2023 Feb 18;13:2890. doi: 10.1038/s41598-023-30045-9 (PMC9938512; doi:10.1038/s41598-023-30045-9)
Supplement: Supplementary file 1 — Supplementary Information. [file 41598_2023_30045_MOESM1_ESM.pdf]

Supplementary Information

Crystal Structure of the CoV-Y domain of SARS-CoV-2 Nonstructural Protein 3

Yunfeng Li[1,4], Yulia Pustovalova[1,4], Wuxian Shi[2], Oksana Gorbatyuk[1], Sridhar Sreeramulu[3], Harald Schwalbe[3], Jeffrey C. Hoch[1] and Bing Hao[1,*]

[1]Department of Molecular Biology and Biophysics, University of Connecticut Health Center, Farmington, Connecticut 06030, USA;

[2]Photon Sciences, Brookhaven National Laboratory, Upton, NY 11973, USA;

[3]Institute for Organic Chemistry and Chemical Biology, Center of Biomolecular Magnetic Resonance (BMRZ), Goethe University Frankfurt, Frankfurt am Main, Germany.

[4]These authors contributed equally to this study.

*Corresponding author: Bing Hao (bhao@uchc.edu)

This PDF file includes:

Supplementary Figures S1 to S6
Supplementary Tables S1 to S3
Supplementary References

Supplementary Figures

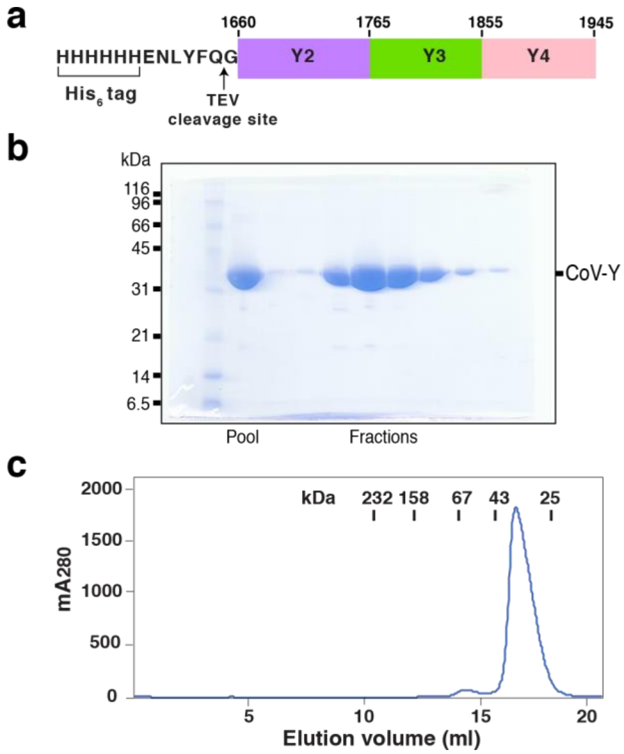

Supplementary Figure S1. Construct design, protein purification and oligomerization state of the nsp3 CoV-Y domain.

a. Schematic representation of the CoV-Y construct used in this study. The amino-acid residue boundaries for each subdomain are indicated. After TEV protease cleavage of the His_6 tag, the final construct contained an artificial N-terminal glycine residue preceding the native CoV-Y sequence.

b. SDS-PAGE analysis of the CoV-Y protein after the final step of purification, stained with Coomassie blue. The pooled sample and the samples of the individual fractions of the size-exclusion chromatography are shown.

c. Size-exclusion chromatography profiles of the purified CoV-Y protein. The retention volumes of proteins of known mass on the Superdex 200 Increase column are indicated. CoV-Y was eluted at a volume of 16.9 ml corresponding to the molecular weight of the monomeric protein at ~32 kDa.

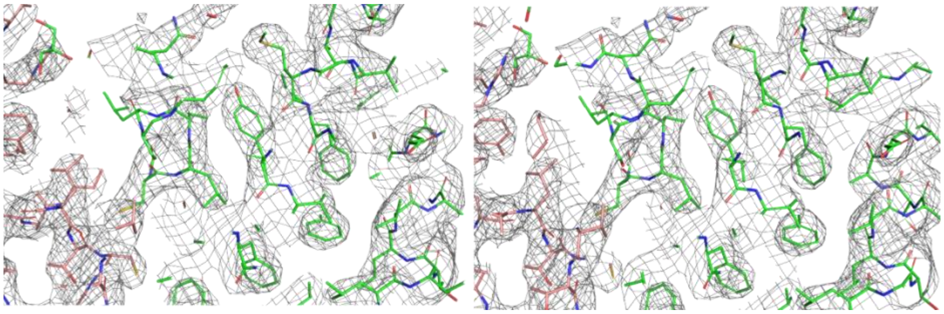

Supplementary Figure S2. Stereo view of a sample of the 2Fo–Fc electronic density map showing the interface of Y2 (in pink) and Y3 (in green).

The residues are shown in stick representation. The map is contoured at 1.0 σ.

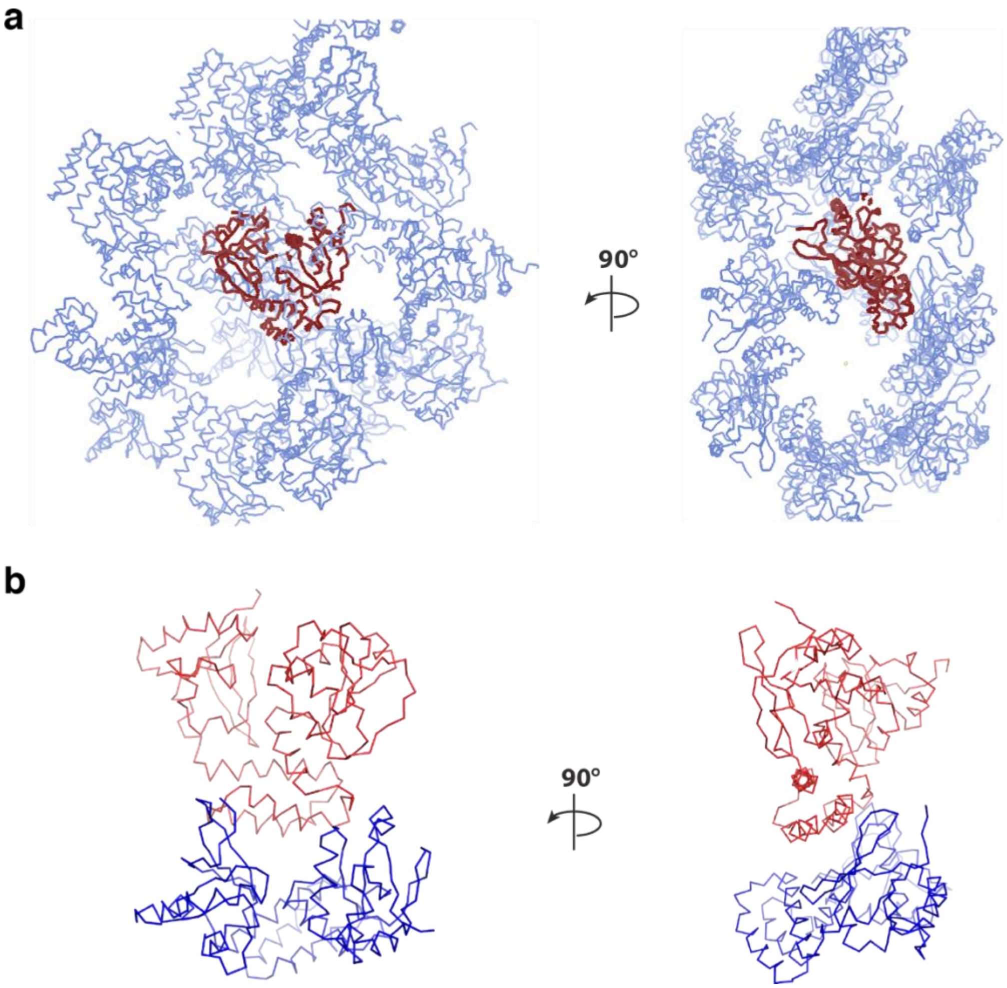

Supplementary Figure S3. Crystal packing-mediated protein contacts in CoV-Y crystal structure.

a. Ribbon representation of CoV-Y (in red) surrounded by the molecules (blue) generated by the crystallographic symmetry.

b. Close-up view of a sample of the crystal-packing contacts between CoV-Y (in red) and a symmetry-related molecule (blue). The association of two molecules buries ~296 Å^2 (~2%) of solvent-accessible surface in each monomer that results in a complex formation significance score (CSS) of 0.0 as calculated by PISA[1]. CSS-score ranges from 0 to 1 indicating that interface relevance to complex formation increases. Therefore, the CSS score of 0.0 implies that the interface shown here does not play any role in CoV-Y assembly and seems to be a result of crysrtal packing only.

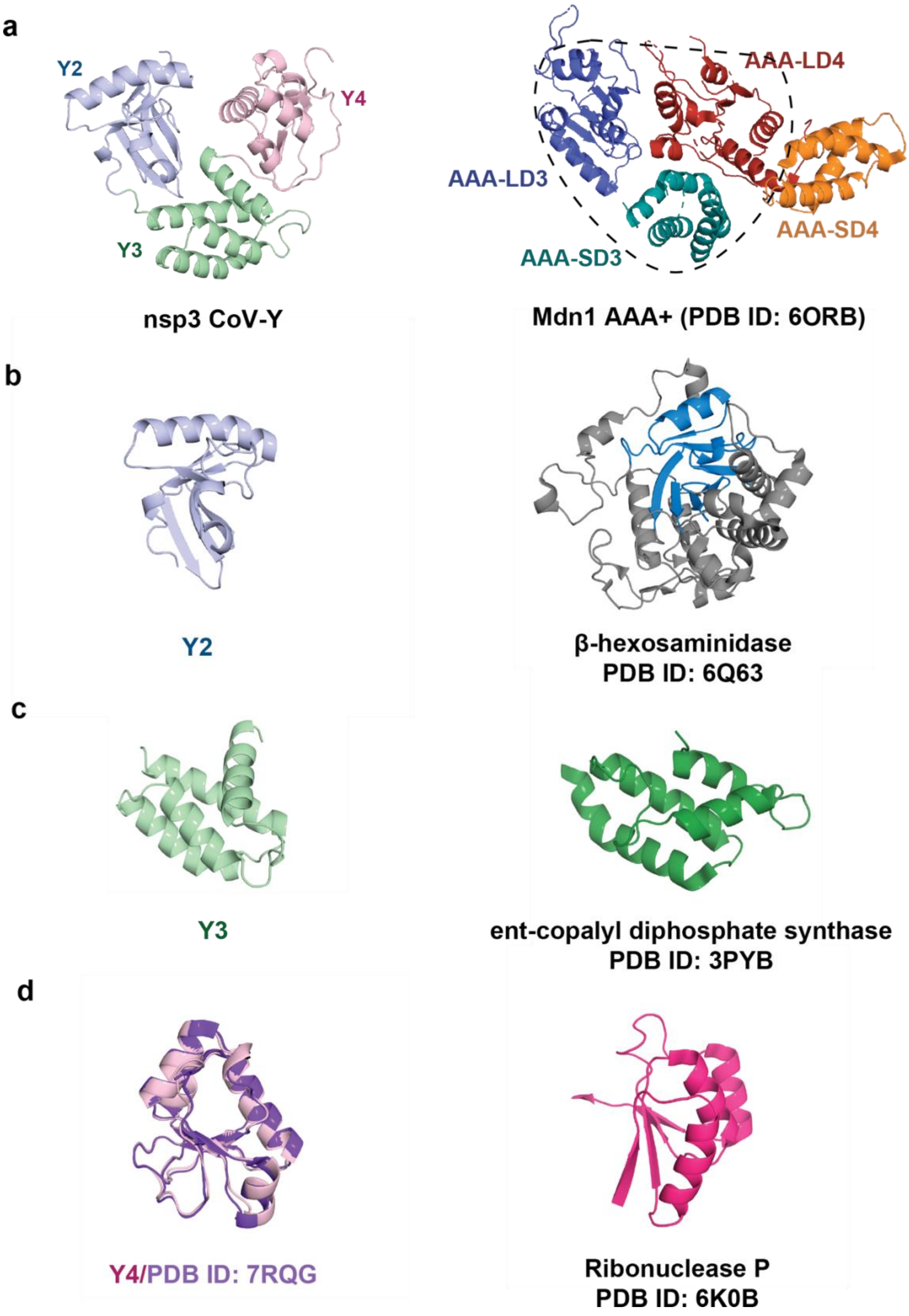
a
Y2
Y4
Y3
nsp3 CoV-Y
AAA-LD4
AAA-LD3
AAA-SD4
AAA-SD3
Mdn1 AAA+ (PDB ID: 6ORB)
b
Y2
β-hexosaminidase
PDB ID: 6Q63
c
Y3
ent-copalyl diphosphate synthase
PDB ID: 3PYB
d
Y4/PDB ID: 7RQG
Ribonuclease P
PDB ID: 6K0B

Supplementary Figure S4. Top results of the Dali searches in PDB for structural similarities between the CoV-Y domain and other proteins.

a. Ribbon diagrams of CoV-Y (left) and a dimer of the AAA-LD and the AAA-SD domains of Mdn1 (right; PDB ID: 6ORB). The region in Mdn1 similar to that of CoV-Y is circled by a dashed line.
b. Ribbon diagrams of the Y2 subdomain of CoV-Y (left) and the N-terminal domain of a *Bacteroides spp.* β-hexosaminidase (right; PDB ID: 6Q63). The region in β-hexosaminidase similar to Y2 is colored in blue.
c. Ribbon diagrams of the Y3 subdomain of CoV-Y (left) and the helical domain of ent-copalyl diphosphate synthase (right; PDB ID: 3PYB).
d. Superimposition of the Y4 subdomain of CoV-Y (left; pink) and the individual Y4 subdomain determined independently (left; purple; PDB ID: 7RQG). The N-terminal domain of archaeal Ribonuclease P (right; PDB ID: 6K0B) was identified by Dali as the one with the highest similarity (Z score) with the Y4 subdomain of CoV-Y following 7RQG.

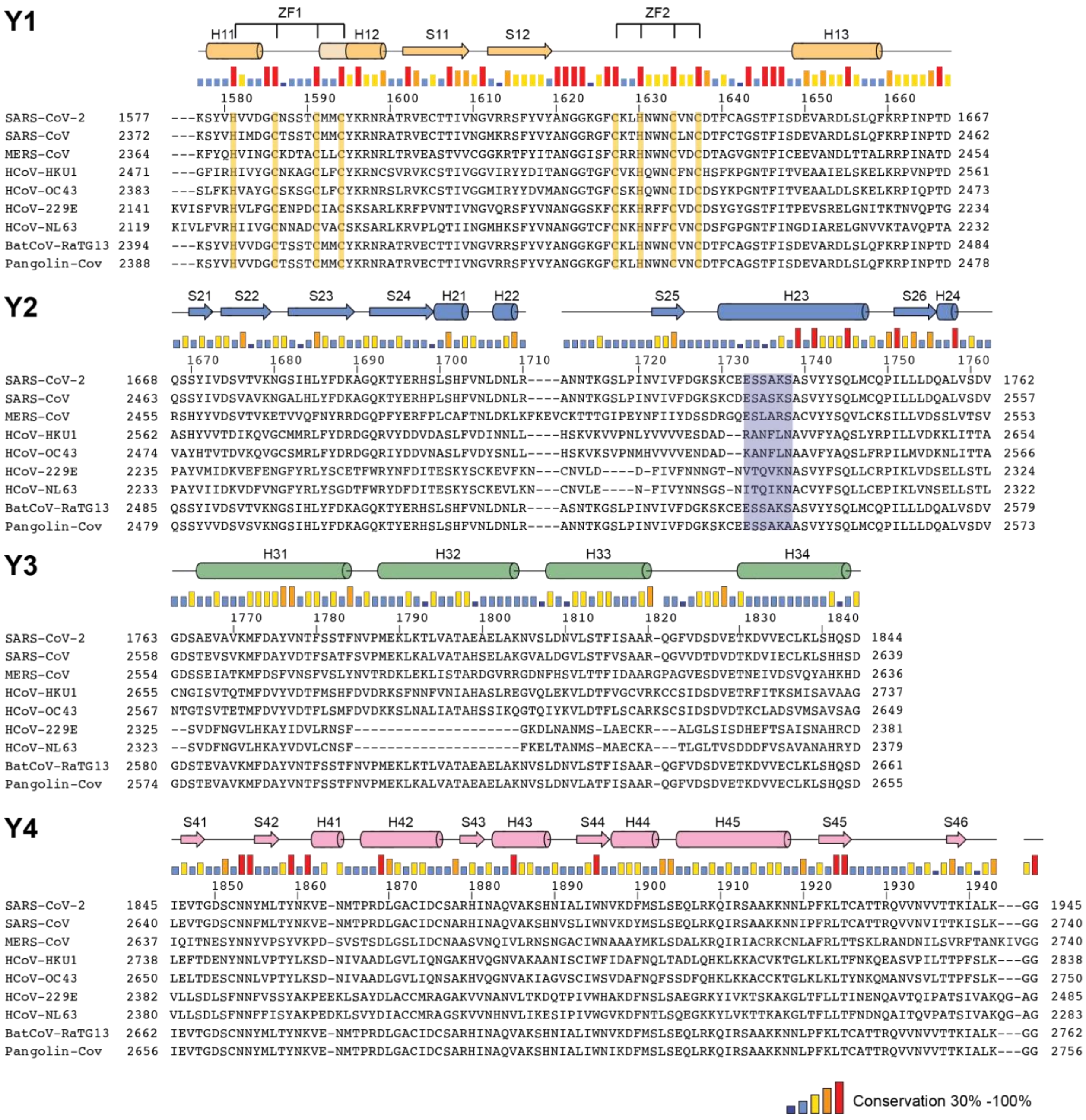

Supplementary Figure S5. Sequence alignment of the nsp3 Y1–CoV-Y homologs.

Sequence conservation is shown as a bar graph, with red bars indicating identity among Y1–CoV-Y homologs. The UniProt accession numbers for each sequence used here are given in the Methods. Secondary-structure elements of Y1 from the computed structural model and CoV-Y from the crystal structure are shown as cylinders (helices) and arrows (β strands). The

coordinating residues of ZF1 and ZF2 of Y1 are highlighted in yellow. The six H23 residues with the most variations in SARS-CoV-2 isolates are highlighted in blue.

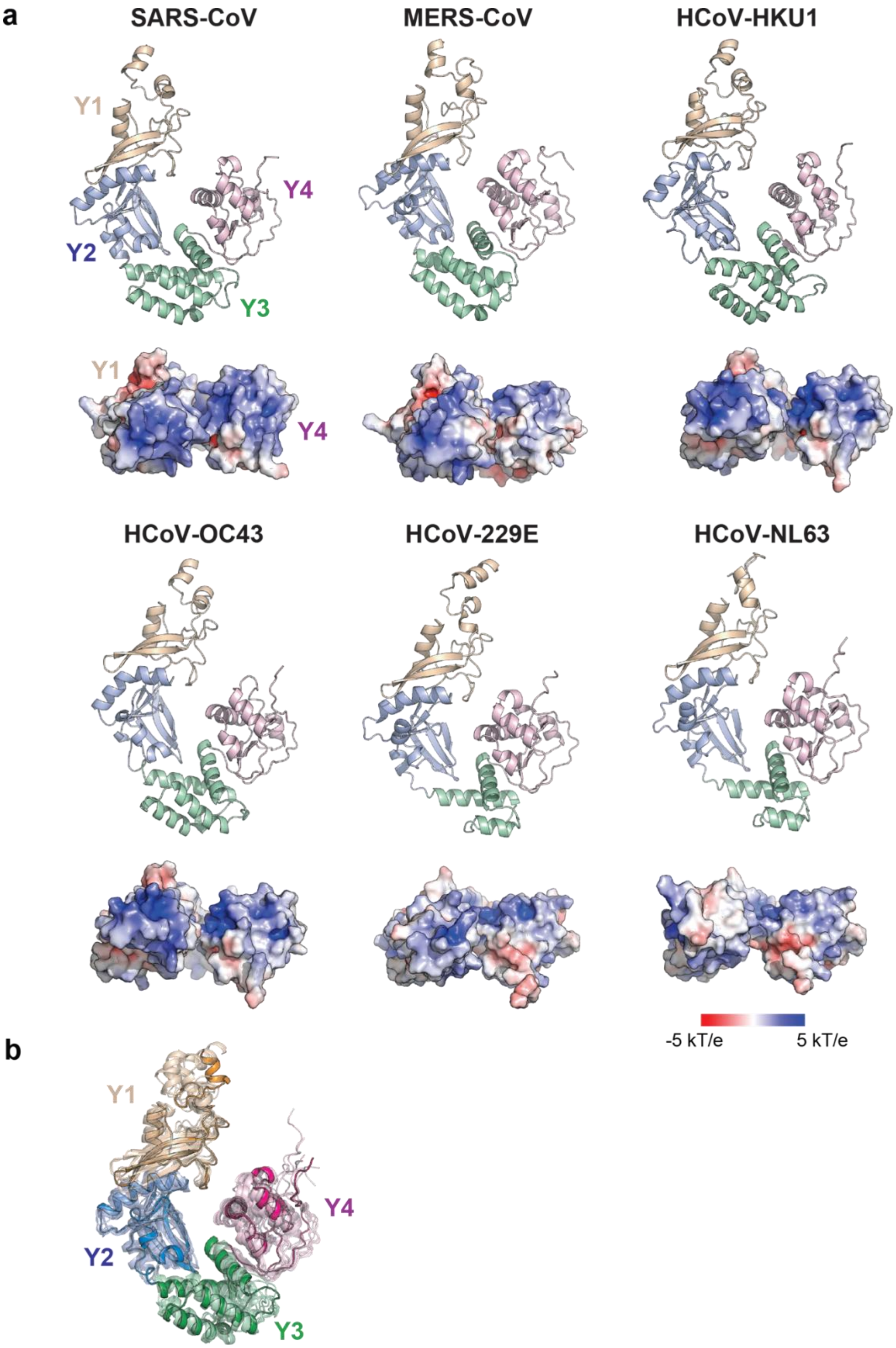
a
SARS-CoV
MERS-CoV
HCoV-HKU1
Y1
Y4
Y2
Y3
Y1
Y4
HCoV-OC43
HCoV-229E
HCoV-NL63
-5 kT/e
5 kT/e
b
Y1
Y4
Y2
Y3

Supplementary Figure S6. Structural conservation among the nsp3 Y1–CoV-Y homologs.

a. Ribbon diagrams of the top-ranked AlphaFold-computed structural models of the nsp3 Y1–CoV-Y domains for four human *Betacoronaviruses* (SARS-CoV, MERS-CoV, HCoV-HKU1 and HCoV-OC43) and two human *Alphacoronaviruses* (HCoV-229E and HCoV-NL63). The three subdomains, Y2, Y3 and Y4, are shown in purple, green and pink, respectively. The corresponding molecular surface representations of the top face of the Y1 and Y4 subdomains from each homolog are presented below the structure and colored according to the local electrostatic potential ranging from -5 kT/e in deep red (most negative) to +5 kT/e in dark blue (most positive) in an orientation similar to that in Fig. 6d.

b. Superposition of the computed SARS-CoV-2 nsp3 Y1–CoV-Y and six structural models from panel a.

Supplementary Table S1. Domain organization and available structural information of SARS-CoV-2 nsp3.

Domains	Domain full name	Residue number range	PDB ID
Ubl1	Ubiquitin-like domain 1	1 – 111	7KAG, 7PKU
HVR	Hypervariable region	112 – 206	–
Mac1	Macrodomain 1	207 – 374	6WEY
Mac2	Macrodomain 2 or SARS-unique domain (SUD)-N	413 – 541	–
Mac3	Macrodomain 3 or SUD-M	549 – 673	7XC4
DPUP	Domain preceding Ubl2 and PL2^{pro} or SUD-C	680 – 743	7P2O, 7THH
Ubl2	Ubiquitin-like domain 2	747 – 802	7THH
PL2^{pro}	Papain-like protease 2	803 – 1050	6Y2E, 6W9C, 7CMD, 6WZU,
NAB	Nucleic acid-binding domain	1089 – 1203	7LGO
βSM	Betacoronavirus specific marker	1230 – 1334	7T9W
TM1	Transmembrane regions 1	1414 – 1436	–
3Ecto	Nsp3 ectodomain	1440 – 1499	–
TM2	Transmembrane regions 2	1500 – 1541	–
Y1	Unknown function domain Y	1576 – 1667	–
CoV-Y	Coronavirus specific unknown function domain Y	1668 – 1945	8F2E*, 7RQG**

* The atomic coordinates and structure factors under this access code will be released upon the publication of the current study.

*Atomic coordinates for the previously determined Y4 subdomain are available under this access code.

Supplementary Table S2. Model variance among the AlphaFold computed CoV-Y structures.

Strains	RMSD to its own top-ranked Model 1 (Å)				
	Model 2	Model 3	Model 4	Model 5	Average
SARS-CoV-2	0.8	0.9	2.4	1.1	1.3 ± 0.7
SARS-CoV	1.3	1.4	2.3	2.0	1.8 ± 0.5
MERS-CoV	3.2	2.6	0.9	2.6	2.3 ± 1.0
HCoV-HKU1	0.8	0.4	0.8	2.5	1.1 ± 0.9
HCoV-OC43	0.8	1.4	2.8	3.0	2.0 ± 1.1
HCoV-229E	1.6	0.8	1.6	1.2	1.3 ± 0.4
HCoV-NL63	0.9	1.0	1.0	0.9	1.0 ± 0.1

Supplementary Table S3. List of the fragments identified as the CoV-Y binder and the binding energy of their top pose as predicted by Autodock Vina[2].

	Name	Chemical formula and SMILE	Structure	Estimated binding energy of the top pose (kcal/mol)
1	2-Benzyloxyaniline	$C_{13}H_{13}NO$ O(C=1C=CC=CC1N)CC=2C=CC=CC2	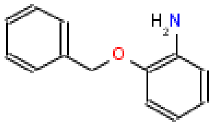	-5.7
2	(4-Methoxyphenyl)(piperidin-4-yl)methanone	$C_{13}H_{13}NO$ O=C(C1=CC=C(OC)C=C1)C2CCNCC2	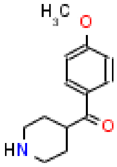	-5.8
3	Methyl 4-[(cyclopentylcarbonyl)amino]benzoate	$C_{14}H_{17}NO_3$ O=C(OC)C1=CC=C(C=C1)NC(=O)C2CCCC2	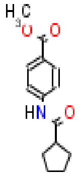	-6.1
4	[4-(1H-1,3-benzimidazol-1-yl)phenyl]methanol	$C_{14}H_{12}N_2O$ OCC1=CC=C(C=C1)N2C=NC=3C=CC=CC32	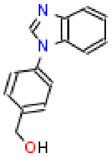	-5.9
5	3-(1,3-thiazol-2-yl)-4H-1,2,4-triazole	$C_5H_4N_4S$ N=1N=C(NC1)C2=NC=CS2	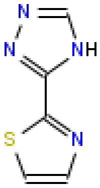	-4.7
6	2-cyclohexyl-N-(4H-1,2,4-triazol-4-yl)acetamide	$C_{10}H_{16}N_4O$ O=C(NN1C=NN=C1)CC2CCCCC2	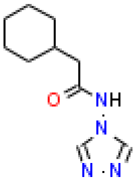	-5.7
7	4-Acetamidophenyl pyrrolidine-1-carboxylate	$C_{13}H_{16}N_2O_3$ O=C(OC1=CC=C(C=C1)NC(=O)C)N2CCCC2	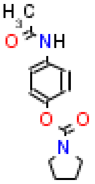	-6.1
8	[1-(4-chlorophenyl)-1H-1,2,3-triazol-4-yl]methanol	$C_9H_8ClN_3O$ ClC1=CC=C(C=C1)N2N=NC(=C2)CO	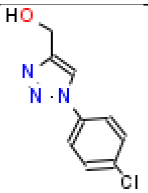	-5.6

9	N,N-Diethyl-p-toluenesulfonamide	C11H17NO2S O=S(=O)(C1=CC=C(C=C1)C)N(CC)CC	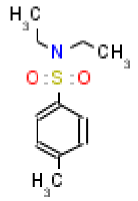	-5.0
10	N-(1H-1,3-benzodiazol-2-ylmethyl)-2-methylpropanamide	C12H15N3O O=C(NCC1=NC=2C=CC=CC2N1)C(C)C	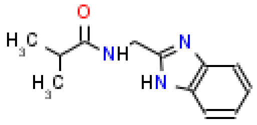	-6.1
11	1-(4-Thiophen-2-ylphenyl)methanamine	C11H11NS S1C=CC=C1C=2C=CC(=CC2)CN	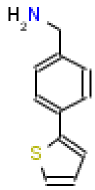	-5.1
12	N-(4-Chlorophenyl)-2-cyanoacetamide	C9H7ClN2O N#CCC(=O)NC1=CC=C(Cl)C=C1	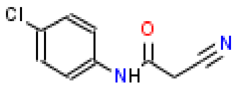	-4.9
13	[1-(3,4-dichlorophenyl)-1H-1,2,3-triazol-4-yl]methanol	C9H7Cl2N3O ClC1=CC=C(C=C1Cl)N2N=NC(=C2)CO	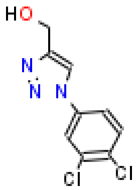	-5.8
14	Ethyl (3-chlorophenyl)carbamate	C9H10ClNO2 O=C(OCC)NC=1C=CC=C(Cl)C1	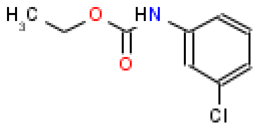	-5.0
15	3-(4-Benzylpiperidin-1-yl)-3-oxopropanenitrile	C15H18N2O N#CCC(=O)N1CCC(CC=2C=CC=CC2)CC1	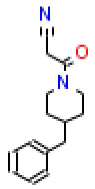	-5.5
16	1-(4-Chloro-phenyl)-3-[1,2,4]triazol-4-yl-urea	C9H8ClN5O O=C(NC1=CC=C(Cl)C=C1)NN2C=NN=C2	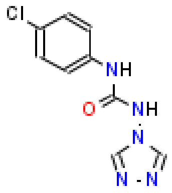	-6.4
17	3-Chloro-4-(4-methylpiperidin-1-yl)aniline hydrochloride	C12H18Cl2N2 Cl.ClC1=CC(N)=CC=C1N2CCC(C)CC2	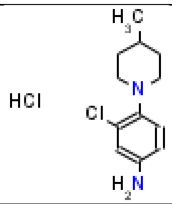	-5.5

Supplementary References

1. Krissinel, E. & Henrick, K. Inference of macromolecular assemblies from crystalline state. *J. Mol. Biol.* **372**, 774–797 (2007).
2. Trott, O. & Olson, A.J. AutoDock Vina: improving the speed and accuracy of docking with a new scoring function, efficient optimization, and multithreading. *J. Comput. Chem.* **31**, 455–461 (2010).
